# Correction: King et al. Establishing an In Vitro System to Assess How Specific Antibodies Drive the Evolution of Foot-and-Mouth Disease Virus. *Viruses* 2022, *14*, 1820

**DOI:** 10.3390/v15020269

**Published:** 2023-01-18

**Authors:** David J. King, Graham Freimanis, Chris Neil, Andrew Shaw, Tobias J. Tuthill, Emma Laing, Donald P. King, Lidia Lasecka-Dykes

**Affiliations:** 1 The Pirbright Institute, Woking GU24 0NF, UK; 2 Department of Microbial and Cellular Sciences, Faculty of Health and Medical Sciences, School of Biosciences and Medicine, University of Surrey, Guildford GU2 7XH, UK; 3 Defence Science and Technology Laboratory (DSTL), Chemical, Biological and Radiological Division, Porton Down, Salisbury SP4 0JQ, UK

## 1. Missing Figure

In the original publication [1], Figure 4 and the corresponding legend were missing. The added Figure 4, showing surface-exposed non-synonymous mutations, appears below. The figure was not included in the original manuscript by mistake, for which the authors would like to apologise.

## 2. Text Correction

There was an error in the original publication. In the Results, Section 3.4, Paragraph 2, sentence “These were located at VP2^66^, VP2^80^ and VP1^155^” should refer to Figure 4. In the Discussion, Paragraph 8, VP^34^ should say VP3^4^.

Corrections have been made to Results, Non-Synonymous Substitutions Exposed on the FMDV Capsid Surface, Paragraph 2, and Discussion, Paragraph 8. Corrected paragraphs appear below.

### 2.1. Results, Section 3.4, Paragraph 2

From the six non-synonymous mutations detected in the region encoding the capsid proteins and described above, three were exposed on the surface of the viral capsid. These were located at VP2^66^, VP2^80^, and VP1^155^ (Figure 4). For VP2^66^, substitution from leucine (L) to phenylalanine (F) led to an accommodation of a larger aromatic side chain, while keeping the site’s hydrophobic properties. Amino acid substitution from lysine (K) to glutamic acid (E) at position VP2^80^ led to a change in the positively charged side chain to a negatively charged side chain. The substitution of alanine (A) to threonine (T) at position VP1^155^ led to a change from a hydrophobic side chain to a polar, uncharged side chain. In both latter cases, the change in the side chain length was only of a single carbon.

### 2.2. Discussion, Section 4, Paragraph 8

In addition to surface-exposed amino acid substitutions, there were three amino acid mutations (L71P in VP4, P4S in VP3, and Q58H in VP1) that were specific to the treatment with the FMDV sub-neutralising sera but found at locations on the surface of the capsid at which they were not predicted to appear. Two of these (at locations VP4^71^ and VP3^4^) were found in the viral population grown in the presence of Challenge Serum 4942, the viral population which showed a reversion in anti-sera-induced delay in the occurrence of CPE through a potential immune escape substitution at VP1^155^. While the N-terminus of the VP4 protein in other picornaviruses is known to be transiently exposed to the outside of the capsid during a process called virus breathing [48,49,50], it is not clear whether the C-terminus of the VP4 protein is exposed to the antibodies. Finally, a single synonymous substitution (S37) was found in the region encoding the VP2 protein. While this substitution was present at a low frequency in the starting inoculum and in samples treated with Control sera, it rose in frequency, reaching the consensus in the viral population passaged in the presence of Field Serum 3159, the viral population which showed a reversion of the delay in time to CPE. Further studies are required to verify the phenotype of these mutations.

The authors state that the scientific conclusions are unaffected. These corrections were approved by the Academic Editor. The original publication has also been updated.

## Figures and Tables

**Figure 4:**
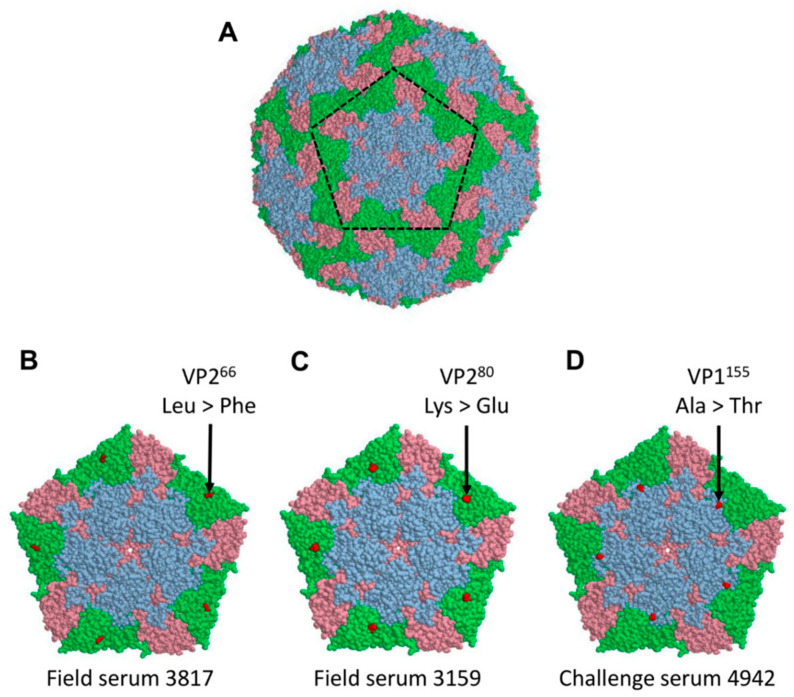
Surface-exposed non-synonymous mutations. The mutations that became fixed at consensus level in the viral populations passaged in the presence of sera from the Field and Challenge groups (but not identified within the starting inoculum or viruses passaged in the presence of the Control sera) were mapped onto the crystal structure of recombinant FMDV A22-H2093F empty capsid. Surface representation of the FMDV capsid (**A**). Individual pentamers enlarged to show mutations identified in the viral population passaged in the presence of Field Serum 3817, Field Serum 3159, and Challenge Serum 4942, respectively (**B**–**D**). The VP2, VP3, and VP1 proteins are highlighted in green, pink, and blue, respectively. Mutations are shown in red.
