# Supplementary material for: Establishing an In Vitro System to Assess How Specific Antibodies Drive the Evolution of Foot-and-Mouth Disease Virus
Source: Viruses. 2022 Aug 19;14(8):1820. doi: 10.3390/v14081820 (PMC9412381; doi:10.3390/v14081820)
Supplement: Supplementary file 1 [file viruses-14-01820-s001.zip › Supplementary Table S1.pdf]

Supplementary Table S1. Sample groups sequenced on separate Illumina MiSeq runs. Each sequencing run contained a starting virus sample and a viral passage series grown in the presence of a Field, Challenged or Control sera.

		Serum groups		
	Starting virus	Field	Challenged	Control
Group 1	One	3157	4926	4926C
Group 2	Two	3817	4914	4914C
Group 3	Three	3159	4942	4942C
