# Supplementary material for: Establishing an In Vitro System to Assess How Specific Antibodies Drive the Evolution of Foot-and-Mouth Disease Virus
Source: Viruses. 2022 Aug 19;14(8):1820. doi: 10.3390/v14081820 (PMC9412381; doi:10.3390/v14081820)
Supplement: Supplementary file 1 [file viruses-14-01820-s001.zip › Supplementary Table S2.pdf]

Supplementary Table S2. The plaque assay PFU, qRT-PCR copy numbers, PCR cycles used and the Qubit quantification of amplified DNA for each virus isolate sample sequence in this study.

ND = Not done

			Plaque assay	qRT-PCR	PCR amplification	
	Sample	Passage	PFU/ml	Copies/µl	PCR cycles	Qubit
Starting Virus	Sample 1	NA	$4.66x10^7$	$1.01x10^8$	26	0.42
	Sample 2			$1.51x10^8$	32	0.71
	Sample 3			$2.30x10^8$	36	2.02
Field Serum	3159	1	$2.95x10^7$	$1.04x10^8$	36	2.19
		2	$2.40x10^7$	$1.20x10^8$	36	1.51
		3	$2.40x10^7$	$2.55x10^8$	36	0.75
		4	ND	$1.07x10^8$	36	1.50
	3157	1	$2.06x10^7$	$2.30x10^8$	26	0.21
		2	ND	$8.81x10^7$	26	0.66
	3817	1	$3.00x10^7$	$1.81x10^8$	32	0.20
		2	$2.57x10^7$	$1.81x10^8$	32	0.19
		3	$3.56x10^7$	$1.55x10^8$	32	0.27
		4	$1.23x10^7$	$1.24x10^8$	32	0.39
Challenged serum	4942	1	$3.10x10^6$	$1.78x10^8$	36	1.88
		2	$5.66x10^6$	$7.09x10^7$	36	2.54
		3	$6.00x10^6$	$1.10x10^8$	36	0.93
		4	$1.33x10^7$	$1.28x10^7$	36	2.94
	4926	1	$6.36x10^6$	$1.38x10^8$	26	0.69
		2	$6.22x10^6$	$8.08x10^7$	26	0.31
		3	$4.50x10^7$	$1.29x10^8$	26	0.76
		4	$2.26x10^7$	$9.17x10^7$	26	0.27
	4914	1	$1.43x10^7$	$1.42x10^8$	32	0.28
		2	$7.90x10^6$	$2.00x10^8$	32	0.61
		3	$3.60x10^7$	$2.08x10^8$	32	0.40
		4	ND	$1.27x10^8$	32	0.49
Control serum	4942C	1	$3.20x10^7$	$2.74x10^8$	36	0.87
		2	$1.73x10^7$	$8.66x10^7$	36	0.20
		3	$3.36x10^7$	$1.48x10^8$	36	32.03
		4	$2.16x10^7$	$9.05x10^7$	36	23.51
	4926C	1	$1.16x10^7$	$1.04x10^8$	26	0.51
		2	$2.23x10^7$	$2.46x10^8$	26	1.54
		3	$4.50x10^7$	$1.68x10^8$	26	1.17
		4	ND	$2.41x10^8$	26	1.27
	4914C	1	$8.00x10^7$	$1.30x10^8$	32	0.36
		2	$2.33x10^7$	$1.82x10^8$	32	0.32
		3	$2.53x10^7$	$1.96x10^8$	32	0.35
		4	$2.36x10^7$	$1.65x10^8$	32	0.33
