# Supplementary material for: Establishing an In Vitro System to Assess How Specific Antibodies Drive the Evolution of Foot-and-Mouth Disease Virus
Source: Viruses. 2022 Aug 19;14(8):1820. doi: 10.3390/v14081820 (PMC9412381; doi:10.3390/v14081820)
Supplement: Supplementary file 1 [file viruses-14-01820-s001.zip › Supplementary Table S3.pdf]

Supplementary Table S3a. Sequence and alignment statistics of each starting virus samples (including technical replicates).

	Sample	Replicate	Total reads	Mapped reads	Mean coverage	Frequency cut-off
Starting virus	Start 1	1	$5.52x10^5$	97.83%	$2.06x10^4$	0.20%
		2	$6.35x10^5$	97.07%	$2.35x10^4$	
	Start 2	1	$2.42x10^5$	98.79%	$8.44x10^4$	0.50%
		2	$2.78x10^5$	98.57%	$9.53x10^4$	
	Start 3	1	$3.42x10^5$	99.10%	$1.29x10^4$	0.50%
		2	$3.61x10^5$	98.86%	$1.36x10^4$	

Supplementary Table S3b. Sequence and alignment statistics of each sample (including technical replicates) within the Field group. Due to the low number of generated reads, sample Field-3817-P2 (highlighted in grey) was removed from downstream analysis.

	Sampl e	Passage	Replicate	Total reads	Mapped reads	Mean coverage	Frequency cut-off
Field	3159	1	1	$4.58x10^5$	98.98%	$1.75x10^4$	0.50%
			2	$4.12x10^5$	98.74%	$1.56x10^4$	
		2	1	$4.60x10^5$	98.87%	$1.74x10^4$	0.50%
			2	$2.27x10^5$	97.86%	$8.15x10^3$	
		3	1	$2.86x10^5$	99.17%	$1.08x10^4$	0.50%
			2	$8.34x10^5$	99.05%	$2.98x10^3$	
		4	1	$3.28x10^5$	98.88%	$1.25x10^4$	0.50%
			2	$4.54x10^5$	98.87%	$1.71x10^4$	
	3157	1	1	$3.67x10^5$	93.38%	$1.31x10^4$	0.20%
			2	$1.20x10^5$	75.20%	$3.28x10^3$	
		2	1	$3.36x10^5$	95.10%	$1.21x10^4$	0.20%
			2	$3.34x10^5$	96.22%	$1.21x10^4$	
	3817	1	1	$1.98x10^5$	98.77%	$6.99x10^3$	0.50%
			2	$2.13x10^5$	98.87%	$7.48x10^3$	
		2	1	$2.25x10^5$	99.22%	$7.96x10^3$	0.80%
			2	$2.31x10^3$	3.45%	$2.64x10^0$	
		3	1	$1.92x10^5$	99.10%	$6.87x10^3$	0.50%
			2	$2.04x10^5$	99.29%	$7.32x10^3$	
		4	1	$2.74x10^5$	99.52%	$9.83x10^3$	0.50%
			2	$3.21x10^5$	99.47%	$1.15x10^4$	

Supplementary Table S3c. Sequence and alignment statistics of each sample (including technical replicates) within Challenged group.

	Sample	Passage	Replicate	Total reads	Mapped reads	Mean coverage	Frequency cut-off
Challenged	4942	1	1	$4.24x10^5$	98.44%	$1.58x10^4$	0.50%
			2	$3.75x10^5$	98.37%	$1.39x10^4$	
		2	1	$4.74x10^5$	98.94%	$1.80x10^4$	0.50%
			2	$3.85x10^5$	98.71%	$1.41x10^4$	
		3	1	$3.39x10^5$	98.73%	$1.28x10^4$	0.50%
			2	$5.52x10^5$	98.84%	$2.08x10^4$	
		4	1	$3.94x10^5$	98.63%	$1.50x10^4$	0.50%
			2	$4.11x10^5$	98.87%	$1.57x10^4$	
	4926	1	1	$5.12x10^5$	96.56%	$1.88x10^4$	0.20%
			2	$3.57x10^5$	96.20%	$1.31x10^4$	
		2	1	$5.35x10^5$	96.42%	$1.98x10^4$	0.20%
			2	$4.83x10^5$	95.36%	$1.76x10^4$	
		3	1	$4.45x10^5$	98.42%	$1.67x10^4$	0.20%
			2	$1.13x10^5$	97.49%	$4.22x10^4$	
		4	1	$3.29x10^5$	94.36%	$1.17x10^4$	0.20%
			2	$4.80x10^5$	96.61%	$1.77x10^4$	
	4914	1	1	$3.39x10^5$	99.22%	$1.21x10^4$	0.50%
			2	$2.40x10^5$	99.23%	$8.47x10^4$	
		2	1	$3.49x10^5$	99.38%	$1.25x10^4$	0.50%
			2	$3.56x10^5$	99.34%	$1.27x10^4$	
		3	1	$2.87x10^5$	99.00%	$1.01x10^4$	0.50%
			2	$2.49x10^5$	99.38%	$8.88x10^4$	
		4	1	$6.19x10^5$	99.16%	$2.26x10^4$	0.50%
			2	$5.51x10^5$	99.06%	$2.02x10^4$	

Supplementary Table S3d. Sequence and alignment statistics of each sample (including technical replicates) within Control group.

	Sampl e	Passag e	Replicate	Total reads	Mapped reads	Mean coverage	Frequency cut-off
Control	4942C	1	1	$3.01x10^5$	99.35%	$1.17x10^4$	0.50%
			2	$2.43x10^5$	99.34%	$0.41x10^3$	
		2	1	$2.80x10^5$	99.02%	$1.08x10^4$	0.50%
			2	$1.75x10^5$	99.49%	$6.74x10^3$	
		3	1	$5.24x10^5$	98.45%	$1.98x10^4$	0.20%
			2	$4.72x10^5$	98.46%	$1.77x10^4$	
		4	1	$3.89x10^5$	98.49%	$1.44x10^4$	0.20%
			2	$2.74x10^5$	98.48%	$1.00x10^4$	
	4926C	1	1	$5.91x10^5$	96.38%	$2.19x10^4$	0.20%
			2	$5.71x10^5$	95.91%	$2.09x10^4$	
		2	1	$4.39x10^5$	97.71%	$1.64x10^4$	0.20%
			2	$4.52x10^5$	98.46%	$1.68x10^4$	
		3	1	$3.70x10^5$	98.31%	$1.39x10^4$	0.20%
			2	$3.69x10^5$	97.88%	$1.38x10^4$	
		4	1	$5.60x10^5$	98.92%	$2.13x10^4$	0.20%
			2	$5.57x10^5$	98.60%	$2.08x10^4$	
	4914C	1	1	$3.24x10^5$	97.67%	$1.10x10^4$	0.50%
			2	$1.16x10^5$	98.63%	$3.92x10^3$	
		2	1	$2.46x10^5$	98.63%	$8.46x10^3$	0.50%
			2	$2.47x10^5$	98.06%	$8.46x10^3$	
		3	1	$3.25x10^5$	97.61%	$1.09x10^4$	0.50%
			2	$2.93x10^5$	98.36%	$1.01x10^4$	
		4	1	$2.28x10^5$	99.21%	$8.20x10^3$	0.50%
			2	$2.01x10^5$	98.72%	$6.92x10^3$	
